# Trends in chloroquine resistance marker, Pfcrt-K76T mutation ten years after chloroquine withdrawal in Tanzania

**DOI:** 10.1186/1475-2875-12-415

**Published:** 2013-11-14

**Authors:** Asia Mohammed, Arnold Ndaro, Akili Kalinga, Alphaxard Manjurano, Jackline F Mosha, Dominick F Mosha, Marco van Zwetselaar, Jan B Koenderink, Frank W Mosha, Michael Alifrangis, Hugh Reyburn, Cally Roper, Reginald A Kavishe

**Affiliations:** 1 Kilimanjaro Christian Medical University College and Kilimanjaro Clinical Research Institute, Moshi, Tanzania; 2 National Institute for Medical Research, Tukuyu Centre, Tukuyu, Tanzania; 3 National Institute for Medical Research, Mwanza Centre, Mwanza, Tanzania; 4 Department of Pharmacology and Toxicology, Nijmegen Centre for Molecular Life Sciences, Radboud University Medical Centre, Nijmegen, The Netherlands; 5 Centre for Medical Parasitology, Department of International Health, Immunology & Microbiology, Faculty of Health and Medical Sciences, University of Copenhagen, Copenhagen, Denmark; 6 London School of Hygiene and Tropical Medicine, London, UK

**Keywords:** *Plasmodium falciparum*, Chloroquine, *Pfcrt*, Tanzania, Drug resistance, Malaria, Mutations, Parasites, Polymorphisms

## Abstract

**Background:**

*Plasmodium falciparum* resistance to anti-malarial drugs remains a major obstacle to the control of malaria. In 2001 Tanzania replaced chloroquine (CQ) with sulphadoxine-pyrimethamine (SP) as first-line drug, which in turn was replaced by artemisinin combination therapy in 2006. SP has however, continued to be used in intermittent preventive treatment of malaria in pregnancy (IPTp) despite reports of high levels of resistance to SP due to the lack of alternatives to SP for IPTp. Recent reports have indicated recovery of CQ-susceptibility in Malawi, Kenya, Mozambique, and Tanzania based on the prevalence of wild types at codon 76 of the *Pfcrt* gene in indigenous *P. falciparum* populations. The current prevalence of this *Pfcrt-*76 CQ resistance marker from six regions of Tanzania mainland is hereby reported.

**Methods:**

DNA extracted from filter-paper dried blood spots and rapid diagnostics kit strips collected from finger-prick blood were used to genotype the *Pfcrt*-76 resistance marker using PCR-RFLP. Data from previously published studies were used to generate CQ susceptibility recovery trends using logistic regression model.

**Results:**

Seven hundred and forty one (741) samples were genotyped. The current frequency of the CQ-susceptible *Pfcrt*-K76 was above 92% and did not differ between regions in Tanzania (*χ*2 = 2.37; p = 0.795). The K76 allelic prevalence was between 85.7 and 93% in regions (*χ*2 = 7.88, p = 0.163). The CQ resistance recovery trends showed regional variability that may be caused by differences in malaria transmission intensity, but overall the trends converge as the susceptibility levels in all regions approach >90%.

**Conclusions:**

CQ withdrawal in Tanzania has resulted into >90% recovery of susceptibility in ten years of withdrawal. These findings are in support of the search for CQ-based combination drugs as a possible future alternative to SP for IPTp in places where full recovery of CQ-susceptibility will be evident.

## Background

Chloroquine (CQ) was the cornerstone of anti-malarial treatment in Africa for almost 50 years but due to resistance in the malaria parasite *Plasmodium falciparum*, CQ was abandoned [1]. Malawi was the first African country to replace CQ in 1993 [2], followed by Kenya in 1998 [3] and Tanzania in 2001 [4].

In Tanzania CQ was replaced by sulphadoxine-pyrimethamine (SP) as first-line treatment and amodiaquine (AQ) as second-line for uncomplicated malaria while quinine remained the third-line for complicated malaria [5]. The CQ policy change was mainly based on *in vivo* efficacy studies that had reported as high as 52% treatment failure by 1999 [5]. Resistance to SP in the East African region had already emerged even before it was declared first-line drug [5-7], thus in Tanzania, SP was an interim solution that lasted for only five years and by the end of 2006 it was replaced with the current artemisinin-based combination therapy (ACT) [8]. Although ACT has been largely adopted by most African countries, SP has continued to be used in intermittent preventive treatment of malaria in children (IPTc) and pregnant women (IPTp). The level of resistance to SP due to its continued use has continued to spread, threatening the future of SP-IPTp [9-12]. This has necessitated an urgent search for an alternative to SP and several options are under evaluation including combinations that include CQ [13].

CQ-resistant falciparum malaria is caused by mutations on two genes, the *P. falciparum* CQ resistance transporter (*Pfcrt*) and multidrug resistance transporter-1 (*Pfmdr1*) both located on the food vacuole of the parasite. While wild-type *Pfmdr1* is thought to transport and accumulate CQ into the parasites food vacuole, mutations N86Y, S1034C, N1042D, and D1246Y abolish this transport leading to reduced CQ-sensitivity reviewed in [14]. Polymorphisms of the mutations have also been linked to resistance or sensitivity to other antimalarials. On the other hand, the CQ transporter *Pfcrt* is a stronger predictor of CQ resistance than *Pfmdr1*. Mutations at codons 72 to 76 leading to amino acids replacements from Cysteine-Valine-Methionine-Asparagine-Lysine (CVMNK) to two major haplotypes Cysteine-Valine-Isoleucine-Glutamate-Threonine (CVIET) prevalent mostly in Africa and Serine-Valine-Methionine-Asparagine-Threonine (SVMNT) in South-East Asia have been associated with CQ resistance [15]. One of the mutations, the *Pfcrt*-K76T, is directly linked with both in-vitro and clinical resistance and is thus used as a biomarker of CQ resistance [16]. Following CQ withdrawal in Malawi, emergency of parasites carrying the CQ-sensitive *Pfcrt-*76 with 100% clinical efficacy was reported just eight years after the policy change [2]. Similar findings have been reported in other countries. Two studies in Tanzania reported restoration of CQ-sensitive *Pfcrt-*76 from 17.1 to 50.7% in five years [17] and from 48 to 89.6% in seven years [18]. In Kenya the restoration was much slower than in Malawi and Tanzania, raising from 5 to 40% in 13 years [3]. This study has investigated the current status of CQ resistance based on *Pfcrt-*76T marker in six regions located in the four major regional zones of Tanzania and estimated the selection coefficients in the regions.

## Methods

Samples used in this study were obtained through collaboration with ongoing studies in six regions of mainland Tanzania between June 2010 and August 2011. Except for the Coastal region where the sample involved pregnant women attending the Kibiti health centre for intermittent preventive treatment of malaria, all other samples were collected from all-age groups. Finger-prick blood on filter paper (Whatman-3) or rapid diagnostic test kits (Mwanza samples only) from febrile patients attending various health facilities in the respective regions were collected after patient’s or children’s guardians had consented to the use of their blood samples for malarial genetic studies. The study sites include Mwanza (Misungwi district) and Kagera (Muleba district) around Lake Victoria in the north-western zone, Tanga (Bondo village) in the northeastern zone, Mtwara (Tandahimba and Mtwara-Urban) and Coastal Region (Kibiti-Rufiji) in the southeastern zone and Mbeya (Kyela and Rungwe districts) in the south-western zone. The malaria-positive rapid diagnostic test (RDT) strips or dried filter-paper blood spots were stored in desiccant at room temperature. Malaria parasite DNA was extracted using chelex-100 method as described previously [19]. Genotyping for *Pfcrt-*K76T was performed using PCR-RFLP methods described by Schneider and others [20]. All PCR reagents and restriction endonucleases were purchased from New England Biolabs (Ipswich, MA, USA). Primers were purchased from Biolegio (Nijmegen, The Netherlands).

Previous publications on *Pfcrt-*K76T in Tanzania were obtained by searching PubMed database with keywords “malaria Tanzania”; “pfcrt Tanzania”, “drug resistance Tanzania” and “chloroquine Tanzania”. Allele frequencies of *Pfcrt-*76 were calculated as the proportion of samples carrying the wild-type form (K76) or mutant form (76T) out of the total of all samples carrying the mutant form only and the wild-type form only. Prevalence was calculated by first adding the number of samples carrying mixed infections to both wild-type only and mutants only, thereby obtaining a new ‘n’ (which includes the mixed infections twice). Prevalence of wild-type and mutant allele was then calculated as the percentage of wild-type plus mixed infection or mutants plus mixed infection out of the new ‘n’.

Comparison of genotype prevalence between regions was performed with a six-sample test for equality of proportions using Pearson's chi-square test statistic. Logistic regression was used to compare trends of decline in prevalence of *Pfcrt-*76T allele and to estimate selection coefficients (s) using R version 2.15.2. To do this, a logistic regression was performed on the data for each region separately. The number of generations per year was taken to be three, as established elsewhere [3]. The s-coefficients are the slopes of the regression lines in the resulting model, and express the proportional change per generation in the ratio of resistant to susceptible alleles. The analysis was performed using R's generalised linear model function with the logit link function and a binomial response. Graphical post-production was performed with Apple Grapher software. The study received ethical approval from the Kilimanjaro Christian Medical University College Ethical Board subsequent to the National IRB (NIMR) approval obtained in the collaborating projects.

## Results

### Prevalence of Pfcrt-K76T in six regions of Tanzania

Seven hundred and forty one (741) samples were genotyped at codon 76 of the *Pfcrt*-gene. Of the total sample set, 672 contained single K76 (susceptible) allele, 42 contained the single 76T (resistant) allele while 27 contained mixed K76/76T (susceptible/resistant) alleles. When mixed infections were excluded, the frequency of the susceptible K76 allele in the regions ranged from 92.1 to 97.1% (Table 1). This distribution was not significantly different between the regions (*χ*2 = 2.38; p = 0.795). Furthermore, when mixed infections were included in calculating allelic prevalence, there were slight differences in prevalence of the susceptible K76 allele between the regions ranging from 85.7% (Mtwara region) to 93.5% (Coastal region) but these differences were again not significant (*χ*2 = 7.88, p = 0.163) (Table 1). Overall these results indicated that the distribution of the *Pfcrt*-K76T resistance marker does not differ significantly between the six regions and that the current prevalence of CQ-susceptible allele is between 85.7 and 93.5%.

**Table 1:** **Distribution of *Pfcrt *** **K76T resistance marker in six regions of Tanzania**

	**Frequency of K76T**				**Prevalence of K76**
**Region**	**K76 (%)**	**76T (%)**	**Mixed**	**n**	**%**
**Tanga**	108 (94.7)	6 (5.3)	2	116	93.2
**Coastal**	130 (93.5)	9 (6.5)	0	139	93.5
**Mtwara**	66 (97.1)	2 (2.9)	3	71	93.2
**Kagera**	82 (92.1)	7 (7.9)	8	97	85.7
**Mwanza**	150 (93.2)	11(6.8)	10	171	88.4
**Mbeya**	136 (95.1)	7 (4.9)	4	147	92.7
**Overall**	672 (94.3)	42 (5.7)	27	741	91

### Comparison of current Pfcrt-76T prevalence with previously published data in Tanzania

**Figure 1:**
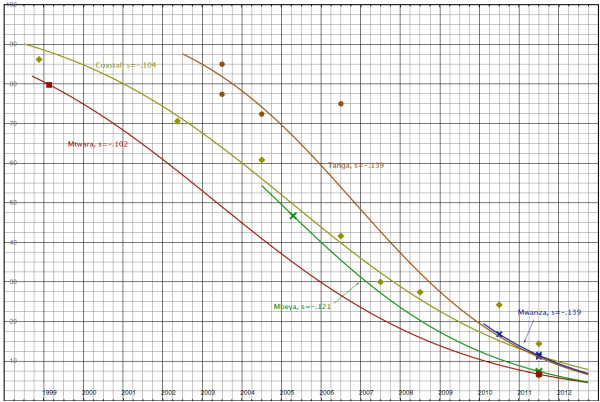
**CQ resistance trends by *Pfcrt*76T between 1999 and 2012 in Tanzania.** Trend lines represent *Pfcrt76T* mutation decrease with time in years; S = selection coefficient; On Y-axis: percentage prevalence; X-axis: years.

A total of eleven papers were retrieved from PubMed. These reports documented frequency of the single K76 or 76T infections and the mixed infections. Allelic frequencies were recalculated from these reports and were used to determine the CQ resistance trends. Table 2 summarizes the data that was used in computing decline in CQ resistance trend lines (Figure 1).

**Table 2:** **Recalculated prevalence of *Pfcrt-*** **76T (mutant *Pfcrt*) from previous studies in Tanzania**

**Region**	**Sites**	**Reported frequency %**	**Mixed**	**Recalculated prevalence (%)**	**Year**	**Number of samples (n)**	**Reference**
Mtwara	Masasi	78.9	13	79.7	1999	71	[20]
Mbeya	Matema and Mbeya urban	43	6	46.7	2005	86	[21]
Coastal	Kibaha	86.2	1	86.2	1998	51	[22]
	Bagamoyo	64.5	16	70.6	2002	76	[23]
		16	29	27.9	2004	175	
	Bagamoyo	52	23	60.8	2004	102	[18]
		28.6	11	41.6	2006	49	
		18.3	43	30	2007	257	
		16.6	28	27.4	2008	187	
		19.4	2	24.2	2010	31	
		11.4	5	14.4	2011	123	
	Bagamoyo	51.9	23	60.8	2004	102	[24]
Tanga	Muheza	82.9	6	85	2003	41	[25]
	Korogwe	70.5	42	76.7	2003	156	[26]
	Korogwe	70.3	42	77.4	2003	155	[17]
		63.1	55	72.4	2004	163	
		67.1	24	72	2007	73	
		49.3	21	75	2006	76	
Mwanza	Igombe	16.8	0	16.8	2010	77	[27]
	Igombe	11.1	0	11.1	2011	90	[28]

Prevalence was recalculated as a percentage of the total when the number of mixed infections is added to both single wild types and mutants.

From the trend lines the following observations are made: (i) the Mtwara and Mbeya trends are based on two data points which are several years apart and should be interpreted with caution; (ii) the Mwanza trend is based on three measurements in a short time span (2010–2011) and generalization of its selection coefficient beyond this period has been done with caution as well; (iii) the Tanga and especially the Coastal regions have well-fitting data across a wide time range. For Coastal region the recalculated prevalence of 27.9% in 2004 [23] (Table 2) was a remarkable outlier, thus was excluded from the analysis; and, (iv) the data used in these trends were collected from different sites within the regions.

Intraregional data from the Tanga and Coastal regions suggest considerable intraregional variation therefore the trends must not be seen as applying to homogeneous regions but rather as general trends within the regions. The trends indicate that decline of CQ resistance started at different moments in different regions, with Mtwara crossing the 80% mark in early 1999, Coastal region at the start of 2001 and Tanga in late 2003. By this time, levels in Coastal and Mtwara were already down to 62%and 47%, respectively. Decline in Tanga, although it started later, was much swifter (s = −0.139) than in Coastal (s = −0.104) and Mtwara (s = −0.102). The first region to fall below the 10% resistant mutants mark was Mtwara in early 2010. The other regions followed and all had crossed the 10% mark by the start of 2012.

### Projections of the Pfcrt-76T prevalence to decrease beyond 1%

Projecting to mid-2013 levels, the current resistance levels could be in the range of 3.5 to 6.3%. However, due to their asymptotic nature, it will not be until late 2016 when the first trend line (Mbeya) is expected to cross the 1% line and mid-2019 when the prevalence of resistance in all regions will be below 1%.

## Discussion

In Tanzania, with the official ban of CQ in 2001, CQ has now been out of use for almost 12 years although self-medication might have continued for a few years later. A survey done in 2002, at about one year post-policy change, reported detection of CQ in only 5% of children aged under-five in Kibaha, Coastal region [29]. At the time of its withdrawal the prevalence of the *Pfcrt-*76T resistance marker is estimated to have been over 80% although this has only been documented in Tanga, Coastal and Mtwara regions (Table 2) [20,22,26]. This study presents the current *Pfcrt-*76T prevalence in six representative regions of Tanzania.

The current frequency of CQ-susceptible *Pfcrt-*K76 marker (over 90%) in all regions and the allelic prevalence beyond 85% in Mwanza and Kagera and over 92% in the rest indicate a rapid decline of the *Pfcrt*-76T marker in Tanzania. While there were no recent data for Mtwara, Mbeya, Tanga and Kagera regions, the results are comparable to the recent findings in Mwanza and Coastal regions where 88.9 and 88.6%, respectively, were reported for *Pfcrt*-K76 in 2011 [18,28].

CQ resistance trends were compared between the regions. The trends have shown a recovery of CQ susceptibility from <20 to >90% in ten years. This trend is comparable to findings in other countries such as Malawi, Mozambique and Kenya. In Malawi recovery of the susceptible *Pfcrt-*K76 from <15 to 100% within 13 years and in Mozambique from <5 to 80% within five years of CQ withdrawal were reported [2,30] while in Kenya a much slower recovery was observed between 1993 to 2006 from 5 to 40%, which is about 13 years after policy change [3]. In Uganda the situation has been very different. Studies conducted in Mulago Hospital, Kampala and Rakai District, southern Uganda, reported between 100 and 98.7% CQ-resistant *Pfcrt-*76T in 2008, about eight years post-CQ replacement due to incomplete CQ withdrawal [31,32], while a recent study in Iganga District, southern Uganda reported 100% resistant *Pfcrt-*76T [27]. Such discrepancies are partly explained by differences in drug policy implementation between countries, although other factors such as differences in malaria transmission patterns and intensity may play a role. Malawi was the first to replace CQ with SP in 1993 followed by Kenya in 1998, Uganda in 1999, Tanzania in 2001 and Mozambique in 2004 [3,4,33,34]. However, in Kenya and Tanzania amodiaquine, a close analogue to CQ, was introduced as second line to SP, while in Uganda CQ was replaced with CQ-SP combination until 2006, when it was replaced by ACT. From 2006 to 2007 a demographic health survey and multiple indicator cluster survey in 21 African countries documented CQ use as 0.8, 0.5, 45.5 and 37% in Malawi, Tanzania, Uganda, and Somalia, respectively [35]. Furthermore, in Rakai District, southern Uganda, a study done in 2007 on “Prescription practices for malaria in rural Uganda” reported prescription of CQ only and CQ + SP at 2.1 and 3.6%, respectively [36]. CQ has been available as home pack CQ-SP formulation for several years in Uganda [37,38]. Likewise in Somalia where CQ was replaced with artesunate + SP combination (AS-SP) in 2005, in 2007 37.7% of public health facilities and 53.1% of private pharmacies were still prescribing CQ as first line [39]. This evidence shows that the lack of synchronized treatment policy across countries has resulted in diversified parasite populations in counties that share the same geographical location.

From these findings, a projected >99% CQ susceptibility by 2019 would be expected under the same conditions that led to the current trends. In Kilifi, Kenya where recovery of CQ susceptibility was much slower, a 100% recovery of CQ susceptibility was predicted by 2026. However, factors such as the declining malaria transmission intensity with consequently reduced drug pressure, introduction of amodiaquine-based ACT and human migration factors may affect such predictions. These findings demonstrate the challenge that African countries have, to achieve the goal of malarial eradication. Now that there is growing evidence for selection of parasites with increased ACT-tolerance [30,40], drugs such as CQ, which is known for its safety, low cost and availability, are good future alternatives for uncomplicated malaria when such drugs are withdrawn for a given period of time. Studies have shown that even in the presence of *Pfmdr1* mutations CQ can remain effective in the absence of *Pfcrt*-76T [2]. However, with the current situation where there is diversified treatment policy across countries, such options may be very narrow. It will be a great challenge to re-introduce CQ in countries where CQ susceptibility has been restored if neighbouring countries still have high levels of resistance. CQ re-introduction will also require regulation of the market against CQ monotherapy. There is a need to harmonize treatment policies across countries or within WHO regions.

Regional differences in selection coefficients were observed although these could not be compared directly. Samples from different sites within the regions were included and correction for intraregional differences in transmission intensity and drug use could not be done. However, regardless of the different regional rates of recovery, the trends clearly are converging, indicating overall homogeneity in the selection pressure for the CQ susceptible marker. This is furthermore supported by the lack of a statistically significant difference in the current prevalence across the country, and is similarly observed in Mozambique where different zones showed different patterns of *Pfcrt-*K76 recovery [34].

Currently there are ongoing trials to determine the use of CQ in combination with other drugs, such as azithromycin [13]. With the currently recommended ACT that has demonstrated effectiveness in clearing multidrug-resistant strains of *P. falciparum*, full recovery of CQ efficacy is a possibility if countries consider withholding CQ use for some years to reverse selection pressure. The WHO has continued to recommend SP-IPTp [41]. The levels of SP resistance have continued to rise and already reports show no or reduced effectiveness of SP-IPTp [10,42]. A recent study in Korogwe, Tanzania reported association of a specific SP-resistant mutation with low birth weight [43]. There is a need for an alternative to SP. Furthermore, as malaria transmission continues to decrease, moving towards elimination will pose the additional challenge of reduced immunity due to reduced malaria exposure in all age groups. This may elevate the chance of pregnant mothers developing clinical disease even at very low parasitaemia, thus requiring effective drugs other than SP for IPTp.

## Conclusions

This report documented more than 90% recovery of CQ susceptibility based on *Pfcrt-*76 biomarker in Tanzania. This is a rapid recovery from the >85% CQ resistance before CQ withdrawal in 2001. This trend has been observed in other countries and provides evidence that removal of drug pressure can result into full recovery of efficacy to drugs that were previous rendered ineffective due to resistance. CQ in combination with another anti-malarial drug remains a promising future alternative to SP in IPTp as a combinational drug if adequate measures are taken to harmonize malaria treatment policy across countries and restrict any continued use of CQ as a monotherapy. Careful evaluation should be done to determine the appropriate CQ combination without reversing the current trends.

## Competing interests

The authors have declared that they have no competing interests.

## Authors’ contributions

AsM performed the experiments, interpreted the data and drafted the manuscript. AN participated in performing the experiments and revised the manuscript. AK, AM, JM and DM supervised sample collection in the field and revised the manuscript. MvZ provided statistical expertise in analysing the data and participated in writing the manuscript. JBK and FWM participated in analysing the data and manuscript writing. MA, HR and CR participated in overall study design and supervision and participated in writing the manuscript. RAK conceived the idea, designed the study, participated in data analysis and wrote the manuscript. All authors read and approved the final version of the manuscript.
